# Inflammatory Cytokines and Clinical Outcome Following Biological Therapy in Adult Bio-Naïve Psoriasis Patients

**DOI:** 10.3390/cimb46070457

**Published:** 2024-07-19

**Authors:** Teodora-Larisa Florian, Ioan-Alexandru Florian, Stefan Cristian Vesa, Lehel Beni, Meda Orăsan

**Affiliations:** 1 Department of Physiology, “Iuliu Hatieganu” University of Medicine and Pharmacy, 400012 Cluj-Napoca, Romania; doratimis@gmail.com; 2 Department of Neurosciences, “Iuliu Hatieganu” University of Medicine and Pharmacy, 400012 Cluj-Napoca, Romania; 3 Department of Pharmacology, “Iuliu Hatieganu” University of Medicine and Pharmacy, No. 23 Marinescu Street, 400337 Cluj-Napoca, Romania; stefanvesa@gmail.com; 4 Department of Pathophysiology, “Iuliu Hatieganu” University of Medicine and Pharmacy, 400012 Cluj-Napoca, Romania; meda2002m@yahoo.com

**Keywords:** psoriasis, inflammatory cytokines, biological therapy, IL-23

## Abstract

Inflammatory cytokines may hold the key to the clinical evolution of psoriasis. The aims of this study are to find a correlation between levels of inflammatory cytokines such as TNF-α, IL-23, IL-17A, and IL-17F and disease duration and severity scores in psoriasis; to test if the decrease in any of the aforementioned cytokines is correlated with an amelioration in disease severity scores; and to analyze if any of the four biologic agents used are linked with a greater decrease in overall cytokine levels. We enrolled 23 adult patients under treatment with ixekizumab, secukinumab, guselkumab, or adalimumab and measured psoriasis disease severity scores PASI (Psoriasis Area Severity Index) and DLQI (Dermatology Life Quality Index), as well as the levels of the aforementioned cytokines at the start of therapy and after 3 months of continuous treatment. Inclusion criteria were the presence of psoriasis, age above 18 years and the need to initiate biological therapy (lack of response to standard treatment). Biological therapies resulted in an amelioration of PASI and DLQI scores, as well as levels of TNF-α, IL-23 and IL-17F. Disease duration and PASI and DLQI scores did not correlate with cytokine levels except DLQI and IL-23 score, in a paradoxically inversely proportional manner. IL-23, in particular, could be a useful biomarker for checking treatment response in psoriasis.

## 1. Introduction

Understanding the complete pathogenesis of psoriasis may lead to the development of a wide array of highly efficient and targeted therapeutic agents. This is especially more pressing as the chronic nature of this disease often translates to recurrences and long-term therapy. However, psoriasis is caused by a plethora of activated immune cells and their respective cytokines, including tumor necrotizing factor alpha (TNF-α), interleukin (IL)-17, IL-22, IL-23, and the granulocyte-macrophage colony-stimulating factor (GM-CSF), which will present elevated levels during the pathophysiological course of the disease [1]. Cytokines play a substantial part in orchestrating and engendering immune cells in psoriasis. Therefore, they could represent ideal therapeutic targets [2].

As a general simplification, psoriasis is a T cell-mediated disease that is reliant on the interaction between the innate and adaptive immune systems [3]. Keratinocytes and dendritic cells (DCs) play critical roles in this process [4]. The initial phase of psoriatic inflammation is followed by a chronic phase that is maintained by positive feedback loops. The precise immune events that initiate the inflammatory cascade are still unknown. The pathomechanism of initiation has been suggested to be autoantigens. The autoantigen that has been the subject of the most research is the cathelicidin antimicrobial peptide, also referred to as LL-37. This peptide is produced by keratinocytes and immune cells in response to skin injury [5,6]. LL-37 activates plasmacytoid DCs (pDCs) via Toll-like receptor-9 (TLR-9) and TLR-7, respectively, by forming complexes with self-DNA or RNA. [7] Consequently, the pDCs generate interferon (IFN)-α, which stimulates conventional DCs (cDCs). Additionally, RNA-LL37 has the potential to stimulate cDCs through TLR8 [8]. Afterward, activated cDCs stimulate and facilitate the expansion of autoreactive T cells by secreting cytokines, including IL–12, IL-23, and tumor necrosis factor TNF-α, and by presenting antigens [9]. The secretion of IL-23, TGF-b, and IL-6 is necessary for the process of differentiation of naïve T cells into T helper 17 cells (Th17), whereas IL-12 is required for the differentiation to Th1. Cytokines are secreted by activated Th cells; Th17 cells secrete IL-17, IL-22, and TNF-α, while Th1 cells secrete IFN-γ and TNF-α. The psoriatic inflammation is exacerbated by the recruitment and stimulation of immune cells by these cytokines, which in turn stimulate the proliferation of keratinocytes and the production of additional inflammatory cytokines in a self-perpetuating vicious circle [3].

TNF-α is the key mediator at the onset of the common form of psoriasis, being also capable of perpetuating the disease in time. It is also involved in T regulator cell inhibition, thereby preventing their action of stimulating the hyperproliferation of pathogenic T cells and of IL-17-producing cells. As a result, the serum levels of TNF-α are significantly increased in psoriasis patients and positively correlated with the PASI score. Dendritic cells release TNF-α and several other cytokines such as IL-23, signaling the formation of CD4+ and CD8+ T cells at the onset of psoriasis. Lastly, T cells migrate toward the superior cutaneous layers, closer to the epidermis [2,10]. Moreover, TNF-α, as a homotrimer cytokine, is associated with cell cycle alteration particularly in keratinocytes and hair follicles in psoriasis. All together, these developments suggest that TNF-α is indeed a suitable biologic candidate for targeted therapy [11].

IL-17 is another appealing target in psoriasis. Characteristically, this cytokine participates in inflammatory cascades and in reconstructing the external cellular barrier. The synthesis of IL-17 induces two symptoms in psoriasis: (1) the proliferation of keratinocytes, and (2) the pathogenic release of cytokines [12]. Regarding the proliferation of keratinocytes, IL-17 induces an aberrant hyperproliferation, with the particular disorganized keratinization. And concerning the release of proinflammatory mediators, it induces the secretion by the keratinocytes of antimicrobial peptides, TNF-α, IL-1b,12 IL-6, IL-36, among others [13].

From the onset of psoriasis down to its self-maintenance systems, IL-23 serves as an integral part of its pathogenesis. Advanced technology using biological agents that inhibit IL-23 has been largely successful, producing encouraging results in psoriasis [14]. Considering its mechanism of action, IL-23 initially forms a complex with its receiver (IL-23/IL-23R). This complex then induces the differentiation of naïve T helper cells into TH17 cells, which subsequently produce IL-17A and IL-17F, the most abundant forms of the IL-17 cytokine. IL-23 also induces the secretion of IL-17, TNF-α and IL-22 by TH-17 cells [15]. Other mechanisms of IL-23 include triggering macrophage proliferation, which will generate an even larger quantum of TNF-α and enhancing the expression of IL-23R, which leads to a vicious circle of self-amplification [2,10].

The purpose of this study was to identify a biomarker that correlates with the more effective response to biological therapy. Thus, we investigated the putative relationships between serum cytokines in psoriasis before and after the initiation of biological therapies, with special attention to intra-individual changes and the correlation with disease severity and quality of life. As such, we looked at whether cytokine levels would have clinical value in assessing disease activity and treatment effects, allowing individualized therapies.

## 2. Materials and Methods

### 2.1. Design and Participants

The following is a longitudinal study on adult, biological-naïve patients with psoriasis. We collected serum from patients who came to our dermatology cabinet between 2018–2021. The inclusion criteria were the presence of psoriasis, the age above 18 years, the quality of being biologically naïve, and the need to initiate biological therapy (lack of response to standard treatment). Exclusion criteria were age of the patient under 18 years, forms of psoriasis that had a favorable response to non-biological therapies, or any prior biological therapy. A total of 23 patients were included in the study, who started biologic treatment with ixekizumab, secukinumab, guselkumab or adalimumab. All patients were naïve in terms of biologics. At the initial visit, patients presented with active psoriasis, displaying extensive skin inflammation. The second visit took place 12 weeks after the initiation of the therapy, with morning fasting samples, in 5 mL tubes without additives. The tubes were left at room temperature for 60 min before centrifuging at 2000 rpm for 10 min, then kept at −80 degrees Centigrade until the samples were analyzed. Elabscience^®^ (Houston, TX, USA) Human TNF-α, Human IL-17A, Human IL-17F, and Human IL-23A ELISA Kits were utilized in measuring serum cytokine levels, in accordance with the instructions provided by the manufacturer. These kits work on the Sandwich-ELISA principle, with the plate provided having been pre-coated with an antibody specific to their respective cytokines. The micro-ELISA plate wells were filled with standards or samples, then the particular antibody is added. Subsequently, each micro plate well was filled with a biotinylated detection antibody specific for the targeted cytokine and an Avidin-Horseradish Peroxidase (HRP) conjugate, which was then incubated, with the free parts then removed by washing. In each well, the substrate solution is added. Using spectrophotometry, the optical density (OD) was measured at 450 nm ± 2 nm wavelength, the cytokine concentration being proportional to the OD. DLQI and PASI scores were evaluated by the treating dermatologist (Orasan Remus-Ioan) at both the first and second visits. The study was conducted in accordance with the Declaration of Helsinki and approved by the Ethics Commission of the University of Medicine and Pharmacy “Iuliu Hatieganu”. Patients had given their informed consent prior to participating in the study.

### 2.2. Statistical Analysis

Data processing was performed with IBM SPSS Statistics, version 21. We analyzed the variation of measured parameters before the start of therapy and at 3 months of treatment. The Chi squared test was used to compare the prevalence of inflammatory syndrome before and after treatment. We used the ANOVA test for repeated measurements, taking into account variables with normal distribution, as well as the Wilcoxon signed rank test for comparing values before and after therapy. The associations in biomarker values and PASI and DLQI scores were tested using the Spearman’s rho correlation coefficient. The limit of statistical significance was considered as *p* < 0.05, and a value of *p* > 0.05 but *p* < 0.1 was considered as a trend towards significance.

## 3. Results

### 3.1. Patients—Values at Start of Therapy

Of the 23 patients included, 14 (61%) were male and 9 (39%) female, for a gender ratio of 1.56:1. 9 patients (39%) came from rural areas, the remaining 14 (69%) from urban areas. The ages ranged from 26 to 63 years, for an average of 51.22 years (SD of 10.66, 95% CI [45.86–54.58]). Initial PASI scores ranged from 17 to 31 points, averaging 24.17 (SD of 3.83, 95% CI [22.61–25.74]), while the DLQI scores had a minimum of 14 points and a maximum of 30 points, the average being 22.52 (SD of 5.83, 95% CI [20.14–24.9]). The Erythrocyte Sedimentation Rate (ESR) values at the start of treatment ranged from 14 to 35 mm/h, with an average of 23.78 mm/h (SD of 5.49, 95% CI [21.54–26.02]). Regarding the C reactive protein (CRP) level, the minimum was 0.7 mg/dL, while the maximum was 12 mg/dL, the average obtained being 3.97 mg/dL (SD of 2.56, 95% CI [3.05–4.89]). A total of 22 (95.7%) patients had inflammatory syndrome before starting biological treatment. Seven patients (30.4%) received ixekizumab, six (26.1%) secukinumab, six (26.1%) guselkumab and four (17.4) adalimumab. The values for cytokines (TNF-α, IL-23, IL-17A and IL-17F) measured at the onset of biological therapy are shown in Table 1.

### 3.2. Inflammation at 3 Months of Therapy

**Figure 1:**
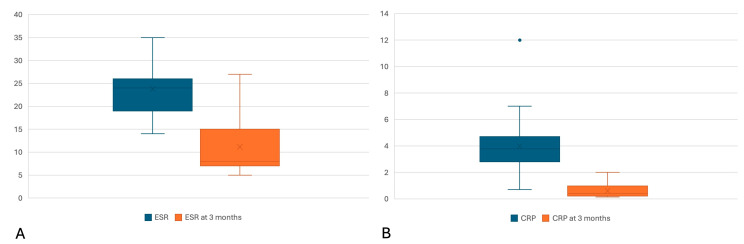
Box plot graphs for the following: (**A**) red blood cell sedimentation rate (ESR) at the onset of biological therapy (blue) and at 3 months of treatment (orange); (**B**) C-reactive protein (CRP) at the start of biological therapy (blue) and 3 months of treatment (orange), respectively. According to the Wilcoxon signed rank test, the differences between each of the 2 measurements were statistically significant (*p* < 0.000). Ordinate: (**A**) the value of ESR in mm/h; (**B**) CRP value in mg/dL. Dots represent outliers.

After three months of biological therapy, the number of patients with serum inflammation markers was eight (38.4%). The ESR values were in the range 5–27 mm/h, the average being 11.17 mm/h (SD of 6.71, 95% CI [8.43–13.91]). Regarding CRP, the serum level varied between 0.14 and 2 mg/dL, with an average of 0.6 mg/dL (SD of 0.51, 95% CI [0.39–0.81]) Using the Chi squared test, the difference between the prevalence of inflammation before and at 3 months of treatment was statistically significant (22 vs. 8, OR 41.25, *p* < 0.0001). Applying the Wilcoxon signed rank test, we observed a significant decrease in the value of ESR (average of differences = −12.61, Z = −4.11, r = −0.9, *p* < 0.000) and CRP (average differences = −3.37, Z = −5.04, r = −1, *p* < 0.000) between the two measurements (Figure 1A,B). Values are shown in Table 2.

### 3.3. Disease Severity at 3 Months of Therapy

Following biological therapy, PASI score values ranged from 3 to 15 points with an average of 9.83 (SD of 3.38, 95% CI [8.45–11.21]). Regarding the DLQI scores, they were between 0 and 14 points, the calculated average being 5.87 (SD of 4.05, 95% CI [4.22–7.53]). According to the Wilcoxon signed rank test, both PASI and DLQI showed statistically significant decreases (average differences = −14.35, Z = −4.19, r = −0.87, respectively, *p* < 0.000 for PASI, respectively, the average of the differences = −16.65, Z = −4.19, r = −0.87, *p* < 0.000 for DLQI).

### 3.4. Inflammatory Cytokine Levels at 3 Months of Therapy

**Figure 2:**
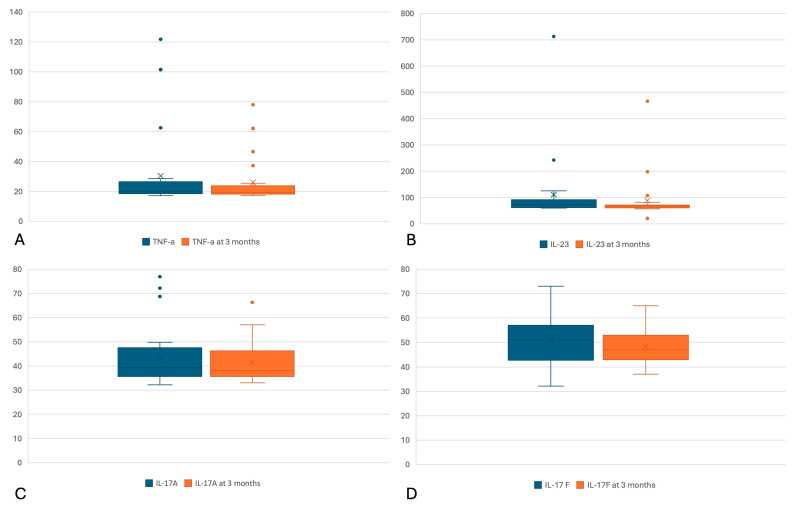
Box plot graphs for (**A**) serum level of tumor necrosis factor alpha (TNF-α) at the start of biological therapy (blue), respectively, and at 3 months of treatment (orange), respectively. According to the Wilcoxon signed rank test, the differences between the 2 measurements showed a trend towards significance (*p* = 0.068 < 0.1). Ordinate: TNF-α level in pg/dL; (**B**) serum level of interleukin 23 (IL-23) at the start of biological therapy (blue), respectively, at 3 months of treatment (orange). According to the Wilcoxon signed rank test, the differences between the 2 measurements were statistically significant (*p* < 0.000). Ordinate: level IL-23 in pg/dL; (**C**) serum level of interleukin 17A (IL-17A) at the start of biological therapy (blue), respectively, at 3 months of treatment (orange). According to the Wilcoxon signed rank test, the differences between the 2 measurements were insignificant (*p* = 0.417). Ordinate: level IL-17A in pg/dL; (**D**) serum level of interleukin 17F (IL-17F) at the start of biological therapy (blue), respectively, at 3 months of treatment (orange). According to the Wilcoxon signed rank test, the differences between the 2 measurements showed a trend towards significance (*p* = 0.098 < 0.1). Ordinate: IL-17F level in pg/dL. Dots represent outliers.

In the next step, we compared the levels of the cytokines TNF-α, IL-23, IL-17A and IL-17F at 3 months from the initial serum levels. According to the Wilcoxon signed rank test, we have achieved significant differences in levels of IL-23 (*p* < 0.000) and a trend towards significance of the difference for both TNF-α (*p* = 0.068) and IL-17F (*p* = 0.098), the values are lower in control. The difference in IL-17A values between 3 months and at onset did not show statistical significance (*p* = 0.42). Table 3 presents cytokine values at 3 months of therapy as well as the results of those comparisons. Figure 2A–D show the differences in cytokines between the two assessments.

### 3.5. Association between Disease Severity and Cytokine Levels

Using the Spearman’s rho test, we checked whether there was a correlation between the duration of the disease in years and the level of cytokines measured. Thus, no statistically significant correlation between disease duration and the four cytokines measured (TNF-α, IL-23, was found, IL-17A and IL-17F) (*p* > 0.05). Similarly, using the same test, a correlation between PASI score and serum proinflammatory cytokine levels could not be identified. Finally, by applying the test for the combinations of DLQI and cytokines, we obtained a significant inversely proportional correlation of moderate intensity between the DLQI and the IL-23 level (rS = −0.479 *p* = 0.021 < 0.05), as well as a trend towards significance for the inversely proportional low-intensity correlation between DLQI and TNF-α level (rS = −0.388 *p* = 0.068 < 0.1). The results are summarized in Table 4.

In the next step, we used the Spearman’s rho to check if there was a correlation between the PASI and DLQI drop on one side and the cytokine levels on the other. In this respect, we obtained a statistically significant correlation between PASI decrease and IL-17A reduction (rS = 0.529, *p* = 0.021 < 0.05), of moderate intensity. Analogously, we found moderate intensity correlations between PASI improvement and IL-23 reduction (rS = 0.368, *p* = 0.084 < 0.1), as well as between the improvement in the DLQI score and the reduction in IL-17A (rS = 0.354, *p* = 0.098 < 0.1) and IL-17F (rS = 0.355, *p* = 0.097 < 0.1). Table 5 presents the results of this test.

### 3.6. Comparison of the Efficiency of Biological Agents in the Studied Group

For the last stage, we used the Wilcoxon signed rank test for each of the biological agents individually, checking for differences between cytokine levels, ESR, CRP, and PASI and DLQI scores. Thus, for ixekizumab, we observed statistically significant differences in the levels of IL-23 (*p* = 0.043 < 0.05) and CRP (*p* = 0.018 < 0.05), respectively, as well as for the improvement in the ESR value (*p* = 0.018 < 0.05) and PASI scores (*p* = 0.018 < 0.05) and DLQI (*p* = 0.018 < 0.05). Similar to secukinumab, the decrease in the IL-23 and CRP levels, ESR and PASI and DLQI scores were statistically significant (*p* = 0.028 < 0.05; *p* = 0.046 < 0.05; *p* = 0.028 < 0.05; *p* = 0.027 < 0.05; *p* = 0.028 < 0.05, respectively). Similarly, guselkumab produced statistically significant differences for the serum level of IL-23 (*p* = 0.046 < 0.05), CRP (*p* = 0.028 < 0.05), and VSH value (*p* = 0.046 < 0.05), PASI score (*p* = 0.027 < 0.05) and DLQI (*p* = 0.027 < 0.05). In the end, we did not achieve statistically significant differences for any of the parameters in the adalimumab-treated group of patients (*p* > 0.05); however, there were trends for statistical significance regarding the reduction in TNF-α (*p* = 0.068 < 0.1), IL-23 (*p* = 0.068 < 0.1), CRP (*p* = 0.068 < 0.1), VSH (*p* = 0.068 < 0.1), PASI score (*p* = 0.066 < 0.1) and DLQI (*p* = 0.068 < 0.1). Table 6 contains the results of this test battery.

Then, we wanted to test whether any of the agents had superior results. By applying the ANOVA test, we did not notice significant differences between the four agents in terms of reducing the level of proinflammatory cytokines (*p* > 0.05). The results of the ANOVA test can be found in Table 7.

## 4. Discussion

The selection of the most effective treatment in psoriasis is often difficult, often consisting of several therapeutic attempts such as the administration of topical therapies initially, followed by the association of systemic therapies and, finally, the transition to biological therapies. From a clinical perspective, it can often be observed that the best results are achieved only with the administration of biological therapies. Most studies in the literature focus on determining and comparing cytokine levels between psoriasis patients and a healthy control group. However, few studies investigate the effects of treatment on cytokine levels over time in the same group of psoriasis patients [16]. Instead, this study focused on cytokine levels in the same individuals over 3 months of therapy, noting disease activity and the effect of biological treatment.

The Choe et al. study demonstrated a significant correlation between PASI score and IL-17A level across the entire patient population, but not with IL-23 [17]. This also applies only to patients with a stable form, but not to patients with an erythrodermic variant of the disease. Contrarily, serum levels of cytokines did not correlate with the severity of the disease or its duration in Bilgic et al.’s study [18]. However, their study showed increased serum levels of the proinflammatory cytokines IL-6, IL-23 and TNF-α in psoriatic patients versus healthy controls. Cataldi et al. did not find a significant impact of age or gender on cytokine levels in psoriatic patients [19]. Using relative quantification, Kutwin and collaborators described an increased gene expression for IL-23A in patients with a PASI score higher than 10 compared to those with a score below this value [20]. This did not apply to the DLQI score. No statistically significant correlations were found between gene expression for IL-17A, IL-17A receptor (IL-17AR), or IL-23 receptor (IL-23R), and PASI or DLQI. This group did not show statistically significant correlations between the assessed cytokine levels and PASI, and between DLQI and IL-23, contrary to expectations, an inverse proportional correlation was noted. This may be due to a statistical error due to the small number of patients included.

After 3 months of treatment with biological agents, the decrease in IL-23 levels was statistically significant, while the reduction in TNF-α and IL-17F tended to be significant. According to Philipp et al., the serum values of IL-17A, IL-17F and IL-22 were significantly reduced from initiation of ustekinumab therapy at weeks 12 and 52 [21]. Similarly, significantly lower values for IL-6 and IL-22 were noted after 36 months of initiation of biological therapies [22]. Therefore, the drop in values may also become significant for interleukins in our study, but only after a longer period of time (12–24 months). In another study conducted by Bramsen Andersen et al., a statistically significant reduction in IL-23, IL-17, was demonstrated, as well as TNF-α 3 months after the administration of biological therapies [23]. However, the patient populations were larger in their study than in the current study, with 131 patients treated with TNF-α inhibitors, 65 with anti-IL-17/agents/IL-17R and 50 patients treated with IL-23 antagonists, respectively. The differences between our study and this may be due to the small number of patients recruited by us (23) compared to the total of 236. In another study demonstrating the efficacy of ustekinumab to reduce cardiovascular risk, the trend toward significance reduction in IL-17A and IL-23 could be seen at 12 weeks (*p* < 0.1) from initiation of therapy, and a more significant reduction in these was achieved at week 52 [24]. Contrarily, anti-TNF-α immunomodulatory treatments did not significantly influence proinflammatory cytokine levels in the Cataldi et al. study [19]. Therefore, regarding the influence of biological therapies on cytokine levels in psoriasis, a number of unknowns remain.

The importance of this study is that it evaluates the dynamic evolution of proinflammatory cytokines before and after biological therapy in psoriasis, in relationship to both clinical amelioration and inflammation reduction. Probably the biggest limitation of this study is the small number of patients included. It is very likely that statistical significance could have been obtained for a larger group of patients. However, the present study clearly demonstrated a significant or trend-to-significance decrease in IL-23, TNF-α and IL-17F, as well as a significant reduction in ESR, CRP, PASI and DLQI scores.

## 5. Conclusions

The present study showed that, in this group of psoriasis patients, biological therapies decrease serum levels of the proinflammatory cytokines TNF-α, IL-23 and IL-17F. Disease duration and PASI and DLQI scores did not correlate with cytokine levels except DLQI and IL-23 score, in a paradoxically inversely proportional manner. IL-23 could be a useful biomarker for checking treatment response. A superior agent as efficacy between ixekizumab, secukinumab, guselkumab or adalimumab could not be identified. In a larger group of patients, statistical significance may be more important.

## Figures and Tables

**Table 1:** Measured cytokine values, inflammatory syndrome parameters, and severity scores in psoriasis at the start of biological therapy.

	TNF-α (pg/mL)	IL-23 (pg/mL)	IL-17A (pg/mL)	IL-17F (pg/dL)	ESR (mm/h)	CRP (mg/dL)	PASI	DLQI
Mean	30.43	110.77	43.63	51.12	23.78	3.97	22.17	22.52
Quartile 25	18.5	61.2	35.7	42.8	19	2.8	21	17
Median	19.7	73.2	39.2	51	24	3.78	24	22
Quartile 75	26.4	91.5	47.5	57	26	4.7	28	29
Min	17.1	60	32.2	32.2	14	0.7	17	14
Max	121.7	713	76.9	73	35	12	31	30
Standard deviation	27.42	136.92	12.52	10.35	5.49	2.26	3.83	5.83
Standard Error	5.71682	28.5502	2.61146	2.15764	1.143734473	0.470460755	0.7993381	1.215625484
95% Confidence	[19.2217569, 41.6312831].	[54.810038, 166.724562].	[38.5077093, 48.7444507]	[46.8928325, 55.3506275].	[21.540922, 26.024278].	[3.04921808, 4.89338992].	[22.6072365, 25.7405835].	[20.1391478, 24.9043122].

Abbreviations: TNF-α, tumor necrotizing factor alpha; IL, interleukin; ESR, erythrocyte sedimentation rate; CRP, C-reactive protein; PASI, psoriasis area severity index; DLQI, dermatology life quality index.

**Table 2:** ESR and CRP values at 3 months of treatment with biological agents and wilcoxon signed rank test results between the 2 measurements (statistical significance was considered for *p* < 0.05).

	ESR at 3 Months (mm/h)	CRP at 3 Months (mg/dL)
Mean	11.17	0.6
Quartile 25	7	0.22
Median	8	0.4
Quartile 75	15	0.98
Min	5	0.14
Max	27	2
Standard deviation	6.71	0.51
Average of differences	−12.61	−3.37
Standard Error	1.399678357	0.106355279
95% Confidence	[8.4305911, 13.9172289]	[0.39372089, 0.81062511]
Z	−4.11	−5.04
r	−0.9	−1
*p*	<0.000	<0.000

Abbreviations: ESR, erythrocyte sedimentation rate; CRP, C-reactive protein.

**Table 3:** Proinflammatory cytokines at 3 months of treatment with biological agents and Wilcoxon signed rank test results between 3 months and start of therapy (statistical significance was considered for *p* < 0.05).

	TNF-α at 3 Months (pg/mL)	IL-23 at 3 Months (pg/mL)	IL-17A at 3 Months (pg/mL)	IL-17F at 3 Months (pg/mL)
Mean	26.04	88.54	41.52	48.53
Quartile 25	18.2	60.6	35.7	43
Median	19.4	67.2	39	47
Quartile 75	23.8	71	48.7	53
Min	17.2	20.6	33	37
Max	78	466.8	66.3	65
Standard deviation	15.66	88.18	8.38	6.81
Standard Error	3.264583129	18.38710243	1.747484383	1.420844384
95% Confidence	[19.6450049, 32.4419351].	[52.5028037, 124.5789163].	[24.5500323, 58.4847477].	[45.7499763, 51.3195837].
Average of differences	−4.38	−22.23	−2.11	−2.587
Z	−1.83	−4.53	−0.81	−1.65
r	−0.38	−0.94	−0.17	−0.36
*p*	0.068	<0.000	0.417	0.098

**Table 4:** Correlations between the levels of circulating inflammatory cytokines, and the duration and severity of the disease before biological therapy.

Cytokine	Coefficient	Disease Duration	PASI	DLQI
TNF-α	r_S_	−0.187	−0.187	**−0.388**
	*p*	0.394	0.394	**0.068**
IL-23	r_S_	0.098	0.098	**−0.479**
	*p*	0.657	0.657	**0.021**
IL-17A	r_S_	0.091	0.091	0.063
	*p*	0.681	0.681	0.775
IL-17F	r_S_	−0.109	−0.109	0.077
	*p*	0.619	0.619	0.728

Abbreviations: PASI, psoriasis area severity index; DLQI, dermatology life quality index; TNF-α, tumor necrotizing factor alpha; IL, interleukin. Bold represents statistical significance or a trend towards significance.

**Table 5:** Correlations between the decrease in level of circulating cytokines and disease severity amelioration after biological therapy.

Cytokine	Coefficient	PASI Decrease	DLQI Decrease
TNF-α decrease	r_S_	0.000	−0.206
	*p*	0.998	0.346
IL-23 decrease	r_S_	**0.368**	−0.091
	*p*	**0.084**	0.679
IL-17A decrease	r_S_	**0.529**	**0.354**
	*p*	**0.010**	**0.098**
IL-17F decrease	r_S_	0.237	**0.355**
	*p*	0.276	**0.097**

Abbreviations: PASI, psoriasis area severity index; DLQI, dermatology life quality index; TNF-α, tumor necrosis factor alpha; IL, interleukin. Bold represents statistical significance or a trend towards significance.

**Table 6:** Wilcoxon signed rank test results for each biological agent, checking the significance of the differences between the values measured at 3 months of therapy from the time of initiation.

Agent	Coefficient	dTNF-α	dIL-23	dIL-17A	dIL-17F	dESR	dCRP	dPASI	dDLQI
Ixekizumab (*n* = 7)	Z	−0.09	−2.03	−1.52	−1.58	−2.37	−2.37	−2.37	−2.37
	*p*	0.933	0.043	0.128	0.115	0.018	0.018	0.018	0.018
Secukinumab (*n* = 6)	Z	−1.15	−2.2	−0.41	−1.49	−2.2	−1.99	−2.21	−2.2
	*p*	0.249	0.028	0.686	0.136	0.028	0.046	0.027	0.028
Guselkumab (*n* = 6)	Z	−0.31	−1.99	−0.42	−0.21	−1.99	−2.2	−2.21	−2.21
	*p*	0.753	0.046	0.674	0.83	0.046	0.028	0.027	0.027
Adalimumab (*n* = 4)	Z	−1.83	−1.83	−0.37	−0.37	−1.83	−1.83	−1.84	−1.83
	*p*	0.068	0.068	0.715	0.715	0.068	0.068	0.066	0.068

Abbreviations: n, number of patients; dTNF-α, the difference between 3 months and the initial tumor necrotizing factor alpha (TNF-α); dIL-23, the difference between 3 months and the initial value of interleukin 23 (IL-23); dil-17A, the difference between 3 months and the initial value of interleukin 17A (IL-17A); dIL-17F, the difference between 3 months and the initial value of interleukin 17F (IL-17F); dESR the difference between 3 months and the initial value of the erythrocyte sedimentation rate (ESR); dCRP, the difference between 3 months and the initial value of C-reactive protein (CRP); dPASI, d, difference between 3 months and baseline psoriatic area severity index (PASI); dDLQI, difference between 3 months and baseline value of dermatology life quality index (DLQI).

**Table 7:** ANOVA test results for comparing the effectiveness between biological agents in reducing the level of proinflammatory cytokines.

ANOVA						
		Sum of Squares	df	Square of Means	F	*p*
TNF-α decrease	Between agents	815.9	3	271.97	1.741	0.193
	Same agent	2967.7	19	156.2		
IL-23 decrease	Between agents	8753.8	3	2917.95	1.112	0.369
	Same agent	49,850.99	19	2623.74		
IL-17A decrease	Between agents	9435.21	3	3145.07	0.814	0.502
	Same agent	73,448.34	19	3865.7		
IL-17F decrease	Between agents	103.28	3	34.43	0.612	0.616
	Same agent	1068.95	19	56.26		

Abbreviations: TNF-α, tumor necrotizing factor alpha; IL, interleukin.

## Data Availability

Data is contained within the article and upon request from the authors.
